# An Applicable Inflammation-Joined and Nutrition-Related Prognostic Indicator in Patients With Colorectal Cancer

**DOI:** 10.3389/fonc.2021.644670

**Published:** 2021-06-17

**Authors:** Guo Wu, Jungang Liu, Haizhou Liu, Lan Jin, Xiaoliang Huang, Xianwei Mo, Huage Zhong, Yanhua Li, Yawei Zhang, Weizhong Tang

**Affiliations:** ^1^ Department of Gastrointestinal Surgery, Guangxi Medical University Cancer Hospital, Nanning, China; ^2^ Guangxi Clinical Research Center for Colorectal Cancer, Nanning, China; ^3^ Department of Environmental Health Sciences, Yale School of public Health, New Haven, CT, United States; ^4^ Department of Experimental Research, Guangxi Medical University Cancer Hospital, Nanning, China; ^5^ Obstetric and Gynecologic Department, West China Second University Hospital of Sichuan University, Chengdu, China; ^6^ National Cancer Center/National Clinical Research Center for Cancer/Cancer Hospital, Chinese Academy of Medical Sciences and Peking Union Medical College, Beijing, China

**Keywords:** colorectal cancer, C-reactive protein, mean corpuscular volume, clinical intervention, risk stratification

## Abstract

**Purpose:**

This study aimed to elucidate the prognostic significance of a novel inflammation-joined and nutrition-related clinicopathological marker for colorectal cancer (CRC).

**Methods:**

Various factors from preoperative fasting blood samples from 2471 patients with CRC were retrospectively analyzed. Factors related to prognosis were evaluated using univariate and multivariate analyses. The Kaplan–Meier method was used to generate survival curves, while the log-rank test was used to measure survival differences between groups.

**Results:**

Univariate analysis revealed that C-reactive protein (CRP)/mean corpuscular volume (MCV) ratio, TNM stage, differentiation, right-sided tumor, age, carcinoembryonic antigen (CEA) level, and CRP level were significantly associated with poor prognosis in CRC. In contrast, adjuvant chemotherapy is regarded as a protective factor. Elevation of CRP/MCV ratio (odds ratio [OR]: 1.535, 95% confidence interval [CI]: 1.121–2.104, P = 0.008), TNM stage (OR: 2.747, 95% CI: 2.175–3.469, P < 0.001), and differentiation (OR, 1.384; 95% CI, 1.150–1.666; P = 0.001) were prognostic risk factors in the multivariate analyses. Subgroup analysis showed that CRP/MCV, TNM staging system, and differentiation also independently affected survival in patients with lymph node-positive CRC. The nomogram based on these three indicators showed that CRP/MCV had a greater prognostic value and clinical significance for lymph node-positive patients with poorly differentiated tumors at the late stage.

**Conclusion:**

A novel nomogram using the clinicopathologic index of inflammation and nutrition was constructed to predict the prognosis of CRC. Early interventions should be emphasized for advanced-stage patients with severe inflammation and poor nutritional status.

## Introduction

Colorectal cancer (CRC) is a major health problem worldwide and the third most common cancer and the second leading cause of cancer-related deaths (1). CRC is estimated to be the second most common cancer in China and ranks as the fifth leading cause of cancer-related deaths regardless of age and sex (1). The incidence and mortality rates of CRC vary significantly worldwide. Even for patients of the same stage, the biological behavior of the tumor and the patient’s prognosis are quite different. Colorectal tumors are heterogeneous, and individualized risk stratification helps guide clinical treatment. It is well documented that inflammatory and nutritional status are both important factors affecting tumor development and clinical outcomes (2, 3). The systemic inflammatory response in cancer patients can lead to malnutrition, which can alter immune responses, increasing the risks of postoperative infection and poor wound healing in surgical patients (4). Early identification of patients who are in danger of a hyperinflammatory state and malnourishment is vital to reduce the risk of surgical complications and mortality, improve clinical outcomes, and relieve the financial burden (2).

Tumors that occur in the digestive tract are more closely related to inflammation and nutrition. On the one hand, they are directly stimulated by digestive juice; on the other hand, intestinal microbes participate in the inflammatory response, and chronic inflammation eventually leads to the occurrence of tumors. For example, Helicobacter pylori infection has been shown to be an important risk factor for gastric cancer, and a persistent chronic inflammatory environment resulting from this infection leads to a series of damages in the gastrointestinal tract (5, 6). Accordingly, *Bacteroides fragilis* and *Enterococcus faecalis* settled in the intestine produce enterotoxins and reactive oxygen species that cause oxidative DNA damage, induce inflammation, and damage the epithelial barrier (7). In addition, the main functions of the digestive tract are digestion and absorption. Tumors that occur in the digestive tract are more likely to develop malnutrition, causing symptoms and signs related to malnutrition. Nutrition, inflammation, immunity, and cancer constitute a triangular relationship, and the imbalance of the qualitative and quantitative nutritional intake is directly related to inflammation and immunity of the body, leading to time-dependent functional degradation and indirectly leading to the occurrence and development of cancer (8). However, few studies have combined the two organically to guide the treatment and prognosis of CRC.

Chronic inflammatory conditions associated with carcinogenesis are characterized by various clinicopathological markers. Many previous studies have investigated the role of preoperative systemic inflammatory markers in CRC prognosis, including the Glasgow prognostic score (GPS), neutrophil-to-lymphocyte ratio (NLR), C-reactive protein to albumin (CAR), lymphocyte-to-monocyte ratio (LMR), platelet-to-lymphocyte ratio (PLR), and systemic organ score (SIS) (9–13). However, patients with gastrointestinal tumors tended to show a different degree of nutritional deficiency, and some patients began to see a doctor because of unknown anemia or even cachexia. In general, right-sided colon cancer (RC) has a high prevalence of microcytic anemia due to luminal blood loss (14). Unlike other nutritional indicators, such as albumin and lymphocytes, MCV is more stable with fewer affected factors (15). MCV, a parameter measuring the variation in red blood cell volume and distinguishing the type of anemia, has been used as a host nutrition index to predict the long-term outcomes in patients with different cancers, including esophageal squamous cell carcinoma, non-small-cell lung cancer, gastroesophageal adenocarcinoma, liver cancer, and CRC (16–21). A retrospective analysis from Japan suggested that preoperative anemia, especially microcytic anemia (MCV<80fl), may serve as an easily available predictor of outcome in CRC.

CRP is an acute phase protein (APR) produced by the liver that has long been employed for clinical purposes, and it can influence multiple phases of inflammation by acting proinflammatory and anti-inflammatory roles (22). Its rapidly rising levels have been linked to a variety of diseases, including the prognosis of various tumors (23, 24). Numerous experimental studies have suggested that CRP upregulation is associated with the development of CRC (25, 26). However, few studies have comprehensively evaluated the prognostic value of CRP/MCV in CRC (27). By combining these two accessible factors, CRP and MCV, we propose an applicable inflammation-joined and nutrition-related clinicopathologic marker for overall survival (OS) prediction in colorectal cancer.

## Methods and Materials

### Patients and Study Design

Overall, 2471 consecutive CRC patients who underwent surgery at the Department of Gastrointestinal Surgery of Guangxi Clinical Research Center for Colorectal Cancer between 2004 and 2019 were enrolled. The inclusion criteria were as follows: (a) patients with histologically confirmed CRC; (b) patients who underwent primary tumor resection; and (c) patients who had no treatment prior to the blood test. The exclusion criteria were as follows: (a) familial adenomatous polyposis or hereditary colon cancer; (b) there were no signs of clinical infection such as fever on the day of blood collection (28), and (c) patients with other neoplastic diseases during the same period. Baseline clinicopathologic parameters, including general basic information; past, personal, and family history; preoperative and postoperative blood routine examination; serological markers and inflammation-related indicators; enhanced computed tomography (CT) and MRI; degree of histological differentiation and pathological TNM staging; *KRAS* and *BRAF^V600E^ *mutation status and microsatellite instability (MSI) testing; and preoperative and postoperative adjuvant chemoradiotherapy, were derived from the medical records. We used the Cox regression model to identify mean corpuscular volume (MCV) as a promising nutritional predictor of prognosis, while C-reactive protein (CRP) was used as an inflammatory index. To explore the value of the CRP/MCV ratio in specific subgroups, we performed a subgroup analysis based on different clinicopathologic parameters.

### Clinicopathological Factors and Definition

The CRP/MCV was obtained by dividing the absolute number of CRP by the absolute number of erythrocyte MCV. MSI testing is characterized by defects in mismatch repair genes (MLH1, MSH2, MSH6, and PMS2). Left-sided colon cancer (LC) was defined as a tumor diagnosed from the splenic flexure of the colon, descending colon, sigmoid colon, and rectum, while right-sided colon cancer (RC) included the ileocecum, ascending colon, hepatic flexure of the colon, and transverse colon, which were consistent with other promulgated articles (29, 30). Routine blood tests were performed using a Mindray BC-6900, and CRP levels were determined using an automatic immunoturbidimetric assay. Tumor markers, including carcinoembryonic antigen (CEA), were measured using an ARCHITECTi2000_SR_ automatic electrochemical luminescence instrument and supporting reagents (Abbott Laboratories, Chicago). TNM staging was evaluated based on the 8^th^ edition of the Union for International Cancer Control classification. The normal ranges CRP were 0–5.0 mg/L for indicates and 80.0–100.0 fl for MCV. The cutoff CEA level was 5 ng/mL.

### Statistical Analysis

All statistical analyses were performed using R software (R version 3.6.2; www.r-project.org). Categorical variables were analyzed using the chi-square test or Fisher’s exact test, and continuous variables were analyzed using Student’s t-test. The cutoff of CRP/MCV was 0.06 × 10^–15^ mg/L^2^ based on the exhaustive method (EXM) to optimize selection. Survival curves were constructed using the Kaplan–Meier method and compared using the log-rank test. Both univariate and multivariate Cox regression models were performed using the Kaplan-Meier survival (Version: 3.1-8) package to evaluate hazard ratios (HRs) and confidence intervals (CIs) for survival based on CRP/MCV and other selected clinicopathological factors. Statistical significance was set at P < 0.05.

## Results

### Baseline Patient Data

**Figure 1:**
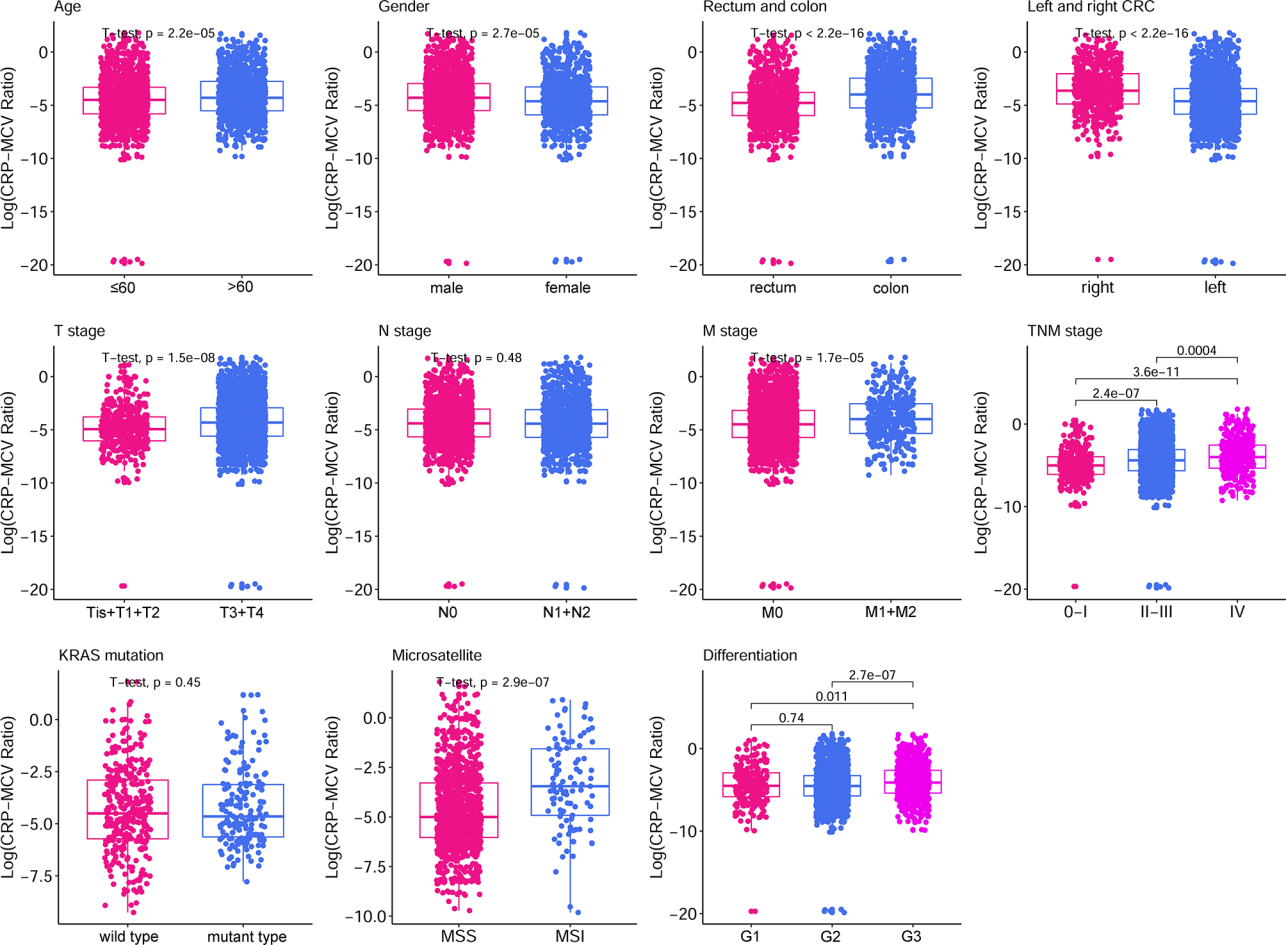
Box plot of clinicopathological features based on CRP/MCV classification. The boxplots show the 5% and 95% confidence intervals. The box plot lower extreme is the first quartile, and the box plot upper extreme is the third quartile. Box plots show the median and whiskers are the minimum and maximum, respectively. Elevated median levels indicated a higher CRP/MCV ratio. The statistical method used for each group was the Student’s t-test. CRP, C-reactive protein; MCV, mean corpuscular volume.

A total of 2471 patients consisting of 1500 (60.7%) men and 971 (39.3%) women were enrolled in this study, and approximately half had rectal cancer. The optimum cutoff value was obtained by considering the correlation of the CRP/MCV ratio with OS in patients. A total of 1468 (59.4%) patients had low risk (CRP/MCV ≤ 0.06) and 1003 (40.6%) patients with high risk (CRP/MCV > 0.06). The male to female ratio was 1.37:1 in the low-risk group compared to 1.86:1 in the high-risk group (*P* < 0.001; **Table 1**). Moreover, the mean age in the low-risk cohort was 57.20 ± 12.67 years, which was lower than that in the high-risk group (59.02 ± 13.16 years; *P* = 0.001) (**Table 2**). The low-risk category tended to have more rectal cancer, and most of them were located on the left side, with 67.6% of cases diagnosed as moderately differentiated. Poorly differentiated, advanced T stage, high probability of metastasis, and microsatellite instability appeared more frequently in the high-risk group. The relationship between the CRP/MCV ratio and baseline clinicopathological characteristics is shown in **Figure 1**. Except for the N stage and *KRAS* status, the increased CRP-MCV was associated with males; older age (older than 60 years); presenting advanced T stage, M stage, and later TNM stage; accompanied by microsatellite instability; and right-sided and poorly differentiated colon cancer.

**Table 1:** Baseline data based on CRP/MCV ratio categorical variables.

Features	Cases	CRP/MCV ratio		χ^2^	*p*
		Low (≤0.0.6)	High (>0.06)		
Total	2471	1468 (59.4%)	1003 (40.6%)		
Sex (%)				13.09	<0.001
Male	1500 (60.7)	848 (34.3)	652 (26.4)		
Female	971 (39.3)	620 (25.1)	351 (14.2)		
Location (%)					
Rectum	1250 (50.6)	873 (35.3)	377 (25.3)	114.14	<0.001
Colon	1221 (49.4)	595 (24.1)	626 (25.3)		
Right	583 (23.6)	239 (9.7)	344 (13.9)	107.29	<0.001
Left	1888 (76.4)	1229 (49.7)	659 (26.7)		
Differentiation (%)				27.05	<0.001
G1	222 (9.0)	130 (5.3)	92 (4.7)		
G2	1576 (63.8)	993 (40.2)	583 (23.6)		
G3	673 (27.2)	345 (14.0)	328 (13.2)		
T stage (%)				28.17	<0.001
Tis+T1-2	401(16.2)	286 (11.6)	115 (4.7)		
T3-4	2070 (83.8)	1182 (47.8)	888 (35.9)		
N stage (%)				0.45	0.50
N0	1362 (55.1)	801 (32.4)	561 (22.7)		
N1-2	1109 (44.9)	667 (27.0)	442 (17.9)		
M stage (%)				19.16	<0.001
M0	2095 (84.8)	1283 (51.9)	812 (31.9)		
M1-2	376 (15.2)	185 (7.5)	191 (7.7)		
TNM stage (%)				53.76	<0.001
0 stage	23 (0.9)	14 (0.6)	9 (0.4)		
I stage	287 (11.6)	213 (8.6)	74 (3.0)		
II stage	938 (38)	520 (21.0)	418 (16.9)		
III stage	847 (34.3)	536 (21.7)	311 (12.6)		
IV stage	376 (15.2)	185 (7.5)	191 (7.7)		
KRAS mutation (%)				0.48	0.49
Wild	323 (13.1)	186 (7.5)	137 (5.5)		
Mutated	171 (6.9)	104 (4.2)	67 (2.7)		
NA	1977 (80)	1178 (47.7)	799 (32.3)		
Microsatellite status (%)				33.67	<0.001
MSI	101 (4.1)	38 (1.5)	63 (2.5)		
MSS	1000 (40.5)	667 (27.0)	333 (13.5)		
NA	1370 (55.4)	763 (30.9)	607 (24.6)		
Neoadjuvant chemotherapy (%)				0.24	0.62
YES	308 (12.5)	179 (7.2)	129 (5.2)		
NO	2163 (87.5)	1289 (52.2)	874 (35.4)		
Postoperative chemotherapy (%)				0.084	0.77
YES	1408 (57.0)	840 (34.0)	568 (23.0)		
NO	1063 (43.0)	628 (25.4)	435 (17.6)		

CRP/MCV, divided C-reactive protein by mean corpuscular volume; MMR, mismatch repair; MSI, microsatellite instability; MSS, microsatellite stability.

**Table 2:** Baseline data based on continuous variables of CRP/MCV ratio.

Features	Cases	CRP/MCV		P
		Low (≤0.06)	High (>0.06)	
Age [year, mean (SD)]	2471	57.20 (12.67)	59.02 (13.16)	0.001
BMI [kg/m^2^, mean (SD)]	2471	22.10 (3.04)	21.96 (3.25)	0.281
CEA [ng/ml, mean(SD)]	2471	14.91 (56.21)	31.91 (108.92)	<0.001
CRP [mg/L, mean(SD)]	2471	2.28 (1.43)	26.43 (34.61)	<0.001
MCV [fl, mean(SD)]	2471	86.57 (9.93)	81.56 (12.17)	<0.001
CRP/MCV [10^-15^mg/L^2^, mean(SD)]	2471	0.03 (0.02)	0.33 (0.44)	<0.001

BMI, body mass index; CEA, carcinoembryonic antigen.

### Univariate and Multivariate Analyses

**Figure 2:**
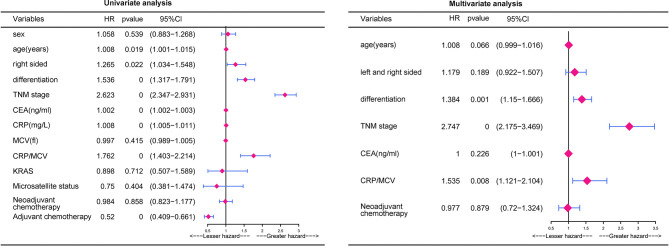
Univariate analysis and multivariate analysis of risk factors for the prognosis in CRC. P value was based on Student’s t-test for continuous factors and Chi-square test (case number ≥ 5) or Fisher’s exact test (case number < 5) for categorical factors.

According to univariate analysis (**Figure 2**), significant differences in cumulative survival were observed for the CRP/MCV ratio together with the TNM stage, differentiation, right side, age, CEA, and CRP levels. Adjuvant chemotherapy was considered to be a protective factor. Sex, MCV level, neoadjuvant chemotherapy, *KRAS*, and microsatellite status did not show significant differences.

**Figure 3:**
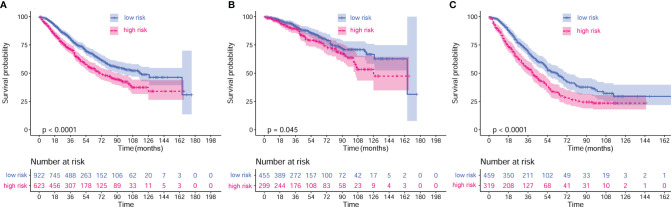
Kaplan–Meier curves of different stages based on CRP/MCV risk stratification. **(A)** Kaplan–Meier curves of all stages based on CRP/MCV risk stratification. **(B)** Kaplan–Meier curves of stage I and II based on CRP/MCV risk stratification. **(C)** Kaplan–Meier curves of stage III and IV based on CRP/MCV risk stratification. A CRP/MCV value above 0.06 indicates high risk and vice versa. The abscissa represents time in months. CRC, colorectal cancer; CRP, C-reactive protein; MCV, mean corpuscular volume.

Multivariate analysis demonstrated that elevated CRP/MCV ratio [odds ratio (OR): 1.535, 95% CI: 1.121–2.104, P = 0.008], TNM stage (OR: 2.747, 95% CI: 2.175–3.469, P < 0.001), and differentiation (OR, 1.384; 95% CI, 1.150–1.666; P = 0.001) were significant predictors of overall survival in patients with CRC (**Figure 2**). Kaplan–Meier curves showed significantly worse survival for patients with high risk than low risk according to the CRP/MCV ratio (**Figure 3**).

### Subgroup Analysis

**Figure 4:**
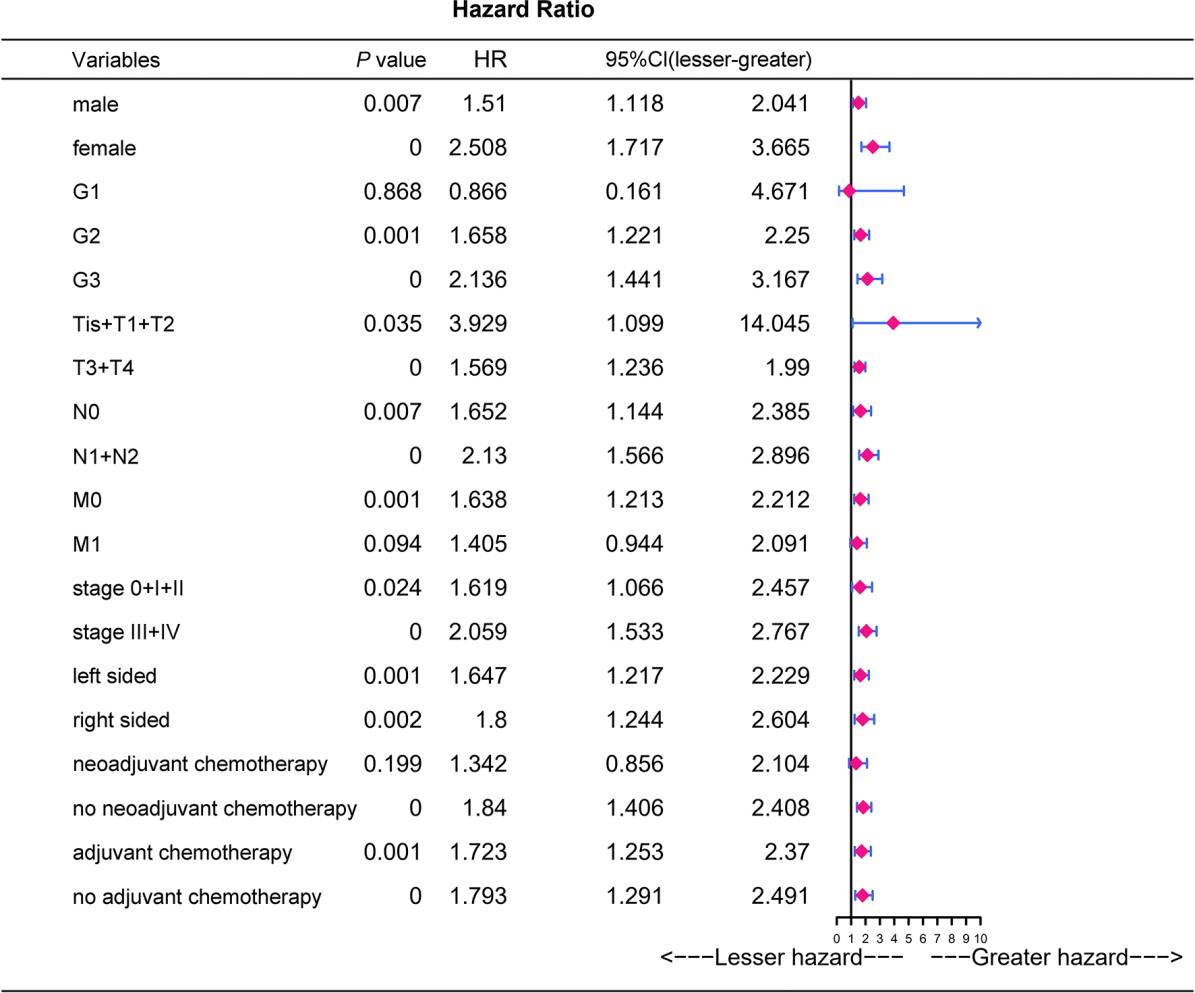
Prognostic analysis of CRP/MCV values in different subgroups. Univariate logistic regression analysis was performed in different subgroups to determine the relationship between CRP/MCV values and prognosis. P value was based on Student’s t-test for continuous factors, and on chi-square test (case number ≥ 5) or Fisher’s exact test (case number < 5) for categorical factors. CRC, colorectal cancer; CRP, C-reactive protein; MCV, mean corpuscular volume.

**Figure 5:**
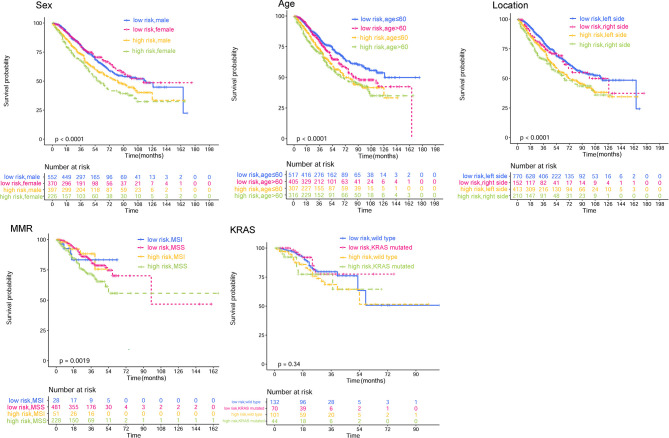
Kaplan–Meier curves of subgroups based on CRP/MCV stratification. The subgroup analysis types are displayed in the upper left corner of the graph, followed by sex, age location, microsatellite status, and KRAS gene type. A CRP/MCV value above 0.06 indicates high risk and vice versa. The abscissa represents time in months. CRP, C-reactive protein; MCV, mean corpuscular volume.

The adverse association of CRP/MCV with overall survival seems to be stronger among women, poor differentiation, early T stage, advanced N stage, advanced TNM stage, and right-side tumor patients (**Figure 4**). As shown in **Figure 5**, the Kaplan–Meier curves in the subgroup analysis expounded the impact of CRP/MCV combined with other clinical features on prognosis. In the sex group, both women and men with CRC in the high CRP/MCV group had a poor prognosis. In the age group, patients with CRC aged > 60 years had a higher CRP/MCV value and worse prognosis. As for the location group, both in the RC and LC cancers had higher CRP/MCV values, which were associated with a worse prognosis. Furthermore, CRC patients with a higher CRP/MCV value diagnosed as microsatellite stable had a worse prognosis than those diagnosed with MSI, which was also found in some favorable randomized control trials (31). The log-rank test suggested that confounding factors affected the prognosis of the KRAS group based on the CRP/MCV ratio (*P* = 0.34, **Figure 5**).

### Subgroup Analysis of Lymph Node-Positive Patients

**Figure 6:**
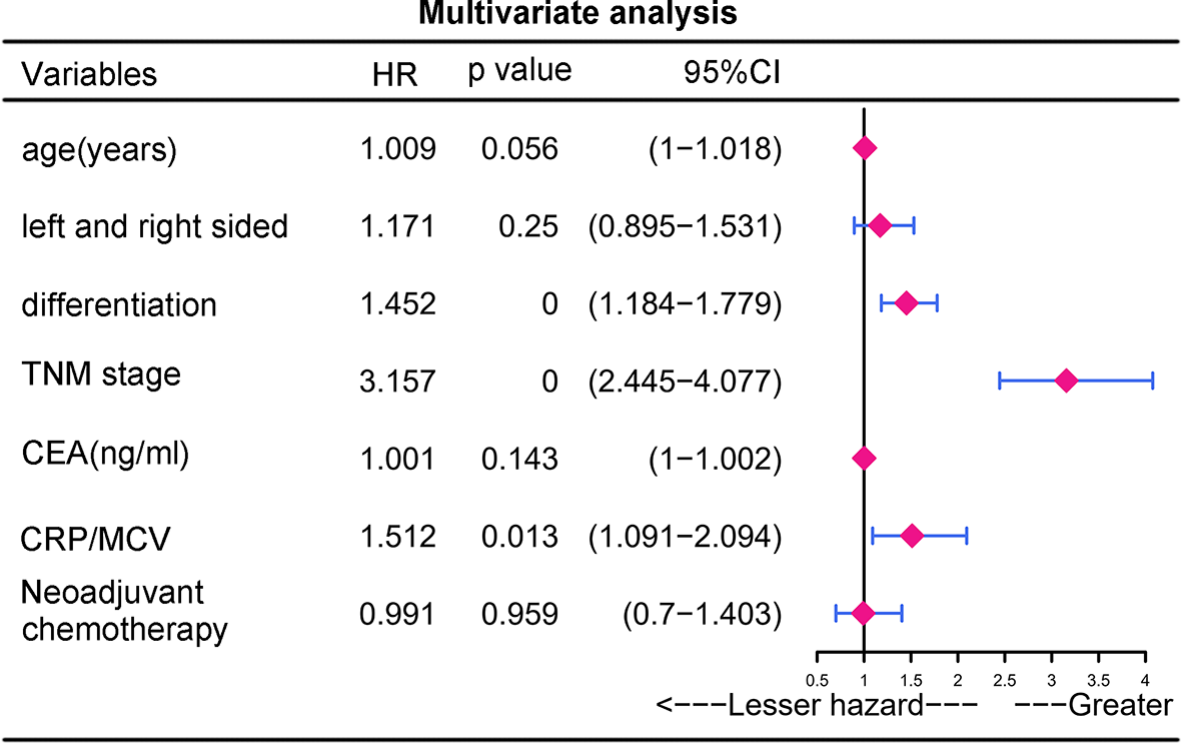
Multivariate analysis of prognostic risk factors for lymph node positive CRC. Multivariate analysis is a multivariate regression analysis of the variables found by univariate analysis.

**Figure 7:**
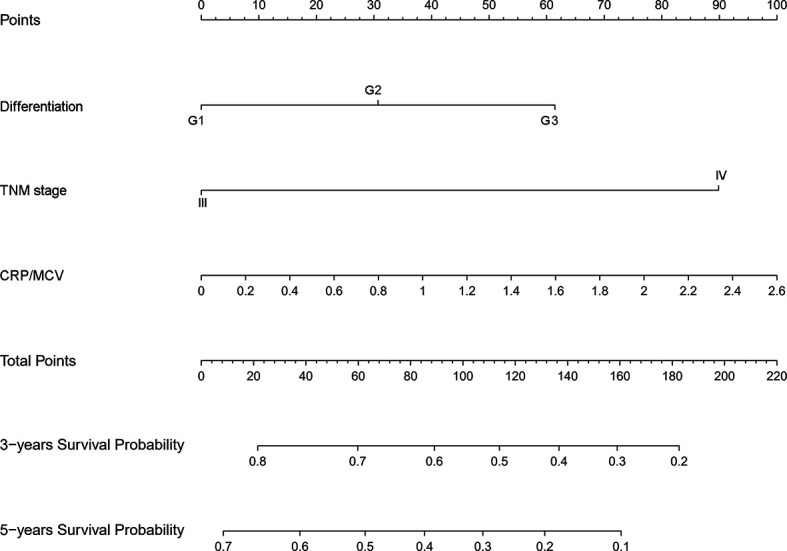
Nomogram for predicting prognosis in lymph node positive CRC. The nomogram was developed in the primary cohort, with the TNM stage, CRP/MCV ratio, and differentiation incorporated.

**Figure 8:**
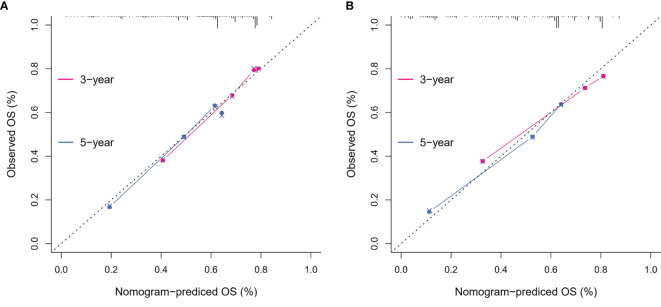
The calibration curves on 3-year and 5-year OS of the nomogram. The x-axis represents the nomogram-predicted probability of overall survival, and the y-axis represents the observed overall survival. The reference line is 45°, which indicates perfect calibration. **(A)** Calibration curves of training set. **(B)** Calibration curves of the validation set.

**Figure 9:**
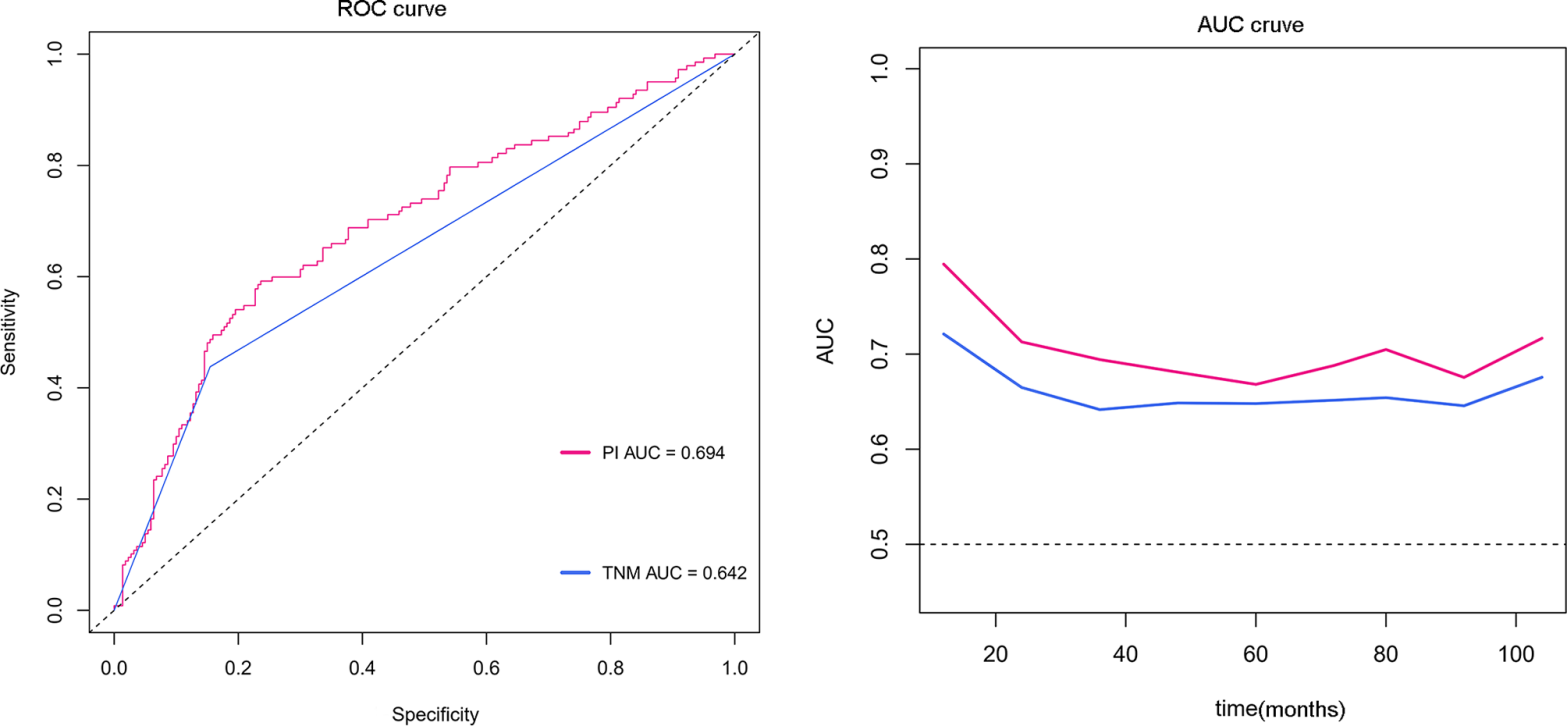
ROC and time-dependent AUC curves for the nomogram. The red line represents the prognostic nomogram, and the blue line represents the TNM staging system alone.

**Figure 10:**
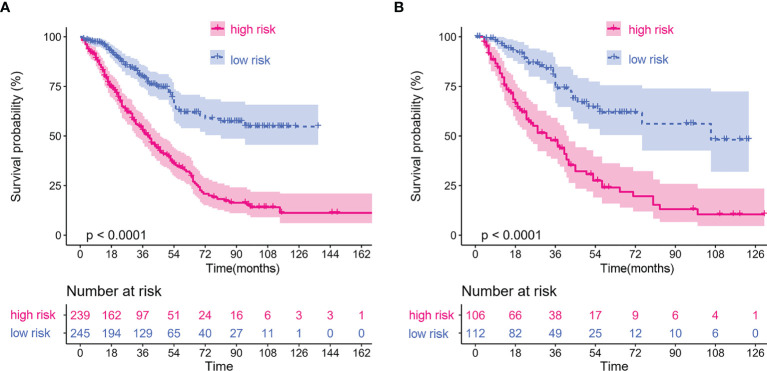
Kaplan–Meier curves of lymph node positive patients based on CRP/MCV stratification. **(A)** The Kaplan–Meier curve of lymph node positive patients in the training dataset. **(B)** The Kaplan–Meier curve of lymph node positive patients in the validation dataset. The abscissa represents time in months.

To identify the subgroups, the relationship between CRP/MCV value and overall survival is stronger, we performed further studies based on the above results. Patients with lymph node-positive CRC were found to have comparable results. Patients (n = 1109) were assigned to a training set (n = 776) and validation set (n = 333). The prognostic nomogram was constructed using a training set and internal verification using the receiver operating characteristic (ROC) curve and the area under the curve (AUC). The goodness of fit between the observed event rates and predicted values was assessed using calibration curves, and Kaplan–Meier curves were used for risk stratification. In multivariate analysis, lymph node-positive CRC patients also demonstrated that elevation of the TNM stage (OR, 3.157; 95% CI, 2.445–4.077; P < 0.001), CRP/MCV ratio (OR: 1.512, 95% CI: 1.091–2.094, P = 0.013), and differentiation (OR: 1.452, 95% CI: 1.184–1.779, P < 0.001) were significant risk factors (**Figure 6**). Adjuvant chemotherapy was considered to be a protective factor. The three independent features listed above were used to construct a prognostic nomogram (**Figure 7**). The predictive nomogram showed that the CRP/MCV ratio was one of the major factors in addition to TNM stage and differentiation. The calibration plot demonstrated a favorable agreement between the predicted and observed values in the primary and validation datasets (**Figure 8**). The ROC curve analysis showed that our nomogram had superior AUC values (0.694) than the TNM staging system alone (0.642) (**Figure 9**), and the model had better and stable predictive values at all times (**Figure 9**). Furthermore, CRP/MCV values significantly distinguished lymph node-positive patient outcomes in both the validation and training sets (**Figure 10**).

## Discussion

This is a retrospective study of clinical big data on laboratory markers for guiding clinical practice in a single center. Among various laboratory indicators, we screened CRP as an inflammation indicator and MCV as a nutritional indicator to form a pioneering prognostic indicator. If the tumor is regarded as “a battle,” CRP can reflect the intensity of the battlefield and the lethality of weapons, while MCV is a fortress against damage. The combination of the two can vividly describe the outcome of a battle.

Since many previous studies have investigated the relationship between inflammation, nutrition, and tumors (8, 32, 33), it is urgent to develop an exact practical clinical index to calculate their prognostic value. CRP plays a role in tumor inflammation, while MCV indicates the level of host nutrition among miscellaneous preoperative indicators. Elevated CRP levels are thought to reflect host reactions to the biological behavior of a tumor (34). The mechanism of CRP upregulation is controlled by proinflammatory cytokines from tumor cells or the immune system, which leads to repeated stimulation and chronic inflammation, forming a carcinogenic microenvironment that favors the development of cancer (35). MCV could serve as an anemia marker that is closely linked with host nutrition, as anemia is caused by tumor bleeding, poor nutrition, and chronic inflammation due to cancer progression (27). A previous report suggested that an insufficient blood supply caused by chronic blood loss and malnutrition induced hypoxia in the tumor microenvironment, leading not only to HIF-1α upregulation but also to T-cell apoptosis, which decreases total lymphocyte levels and contributes to tumor revascularization and proliferation (36). In addition, Nagai et al. showed that in patients with an elevated MCV level, the benefits of 5-FU-based chemotherapy could be predicted by blocking thymidylate synthase (TS), which is involved in DNA synthesis (18). Based on the above facts, we hypothesized that CRP divided by MCV was used as a germane indicator to assess the therapeutic effect and predict long-term outcomes in CRC patients.

In the present study, the CRP/MCV ratio, TNM stage, and differentiation were identified as three independent prognostic indicators in the multivariate analysis. Although the pathological TNM stage was the most important prognostic indicator in patients with CRC, it was not as preoperatively available and dynamically changed to CRP/MCV or CEA. Instead, CRP/MCV can be considered a clinically friendly indicator. Conventional tumor markers such as CEA are well known to be significant indicators of disease burden, posttreatment surveillance, and prognostic value, as they are thought to be secreted from the tumor itself (37, 38). Interestingly, CRP as a prognostic indicator in univariate analysis no longer made sense in the multivariate analysis. When combined with MCV, multivariate analysis revealed that the CRP/MCV ratio was superior to CEA in this respect. Further work is required to evaluate whether a combination of CRP divided by MCV is more valuable for early diagnosis and recurrence compared with CEA.

According to the CRP/MCV classification, the high-risk population was closely associated with men over 60 years of age who presented with advanced T, M, and TNM stages, accompanied by MSI, right-sided colon cancer, and poor differentiation. In other words, preoperative CRP/MCV could predict aggressive tumor biology (39), which is conducive to risk stratification and guiding clinical work. Indeed, estimation of changes in CRP reflects the presence and intensity of an inflammatory process and differentiates inflammatory from non-inflammatory conditions, which are useful in managing the patient’s disease and predicting the prognostic value in certain diseases (40). It has been reported that anemia in CRC frequently shows a microcytic phenotype (39, 41), especially in high-grade T stage, proximal colon tumor location, lymph node metastasis, and elevated serum CRP with or without hypoalbuminemia (14, 27). Based on the above factors, we can speculate that a high level of CRP indicates that the inflammatory response of the tumor is obvious, while low levels of MCV appear in the manner of chronic anemia and poor nutritional status of the host, which leads to increased CRP/MCV values and poor prognosis in patients with CRC. The Kaplan–Meier curves of subgroup analysis showed that the CRP/MCV ratio could also separate the stand or fall of prognosis according to sex, age, location, and microsatellite status, which means that our indicator can easily distinguish a poor prognosis group from a good prognosis group.

Finally, CRP/MCV was also a significant prognostic indicator in patients with positive lymph nodes in the subgroup analysis. Multivariate analysis showed that CRP/MCV was one of the main prognostic factors in addition to the TNM staging system. The nomogram showed that CRP/MCV had more prognostic and clinical significance in lymph node-positive patients with poorly differentiated tumors at the late stage. Within the range of 30–80% of the 3-year survival probability, the corresponding line intervals decreased. For example, when the CRP/MCV value increased by the same amount, patients at stage IV had a lower 3-year survival rate than those with the same differentiation at stage III. Thus, in more advanced patients, it is more difficult to survive in the early 3 years if the inflammatory response is obvious and the nutritional status is poor, which guides clinical interventions. Improving nutrition and reducing inflammation may help advanced cancer patients reach early stages.

This study has several limitations. First, it had a retrospective design and was conducted in a single institution, which could have resulted in selection bias. Second, several diseases, such as iron deficiency anemia, alpha or beta-thalassemia minor, and liver disease, which can affect the value of MCV, were not screened; this may have led to a selection bias. Third, the use of a single parameter to assess nutritional status has been questioned since many other nutritional factors affect outcomes (40). Lastly, this study was conducted over a long period between 2004 and 2019, which can be associated with historical biases in treatment strategy and perioperative management.

In conclusion, a new applicable inflammation-joined and nutrition-related clinicopathologic measurement was constructed to predict the prognosis of patients with CRC. Elevated CRP/MCV can predict the biological behavior of CRC. Severe inflammation and malnutrition suggest poor early prognosis and guide early clinical intervention.

## Data Availability Statement

The raw data supporting the conclusions of this article will be made available by the authors, without undue reservation.

## Author Contributions

GW, JL, HL, and WT contributed to study designs. GW, XM, and HZ contributed to material support and data acquisition. XH, JL, and HL performed the statistical analysis, and GW drafted the manuscript. LJ, LY, and YZ have revised the manuscript. All authors contributed to the article and approved the submitted version.

## Funding

This work was supported by the 2019 Guangxi University High-level Innovation Team, the Project of Outstanding Scholars Program, Guangxi Science and Technology Project (2019AC03004), Guangxi Clinical Research Center for Colorectal Cancer (Guike: AD19245197), Research Basic Ability Improvement Project for Guangxi Young College Teachers (2021KY0087), Innovation Project of Guangxi Graduate Education (YCSW2021133), and Future Academic Star of Guangxi Medical University (WLXSZX21125).

## Conflict of Interest

The authors declare that the research was conducted in the absence of any commercial or financial relationships that could be construed as a potential conflict of interest.
